# Institutional outbreak involving multiple clades of IMP-producing *Enterobacter cloacae* complex sequence type 78 at a cancer center in Tokyo, Japan

**DOI:** 10.1186/s12879-021-05952-9

**Published:** 2021-03-22

**Authors:** Sohei Harada, Kotaro Aoki, Daisuke Ohkushi, Koh Okamoto, Kazumi Takehana, Tomomi Akatsuchi, Keito Ida, Daigo Shoji, Yoshikazu Ishii, Yohei Doi, Kyoji Moriya, Brian Hayama

**Affiliations:** 1 Department of Infection Control and Prevention, The University of Tokyo Hospital, 7-3-1 Hongo, Bunkyo-ku, Tokyo, 113-8655 Japan; 2 Department of Infectious Diseases, Cancer Institute Hospital, Japanese Foundation for Cancer Research, 3-8-31 Ariake, Koto-ku, Tokyo, 135-8550 Japan; 3 Department of Microbiology and Infectious Diseases, Toho University School of Medicine, 5-21-16 Omori-nishi, Ota-ku, Tokyo, 143-8540 Japan; 4 Department of Infectious Diseases, The University of Tokyo Hospital, 7-3-1 Hongo, Bunkyo-ku, Tokyo, 113-8655 Japan; 5 Clinical Laboratory, Cancer Institute Hospital, Japanese Foundation for Cancer Research, 3-8-31 Ariake, Koto-ku, Tokyo, 135-8550 Japan; 6 Department of Infection Prevention, Cancer Institute Hospital, Japanese Foundation for Cancer Research, 3-8-31 Ariake, Koto-ku, Tokyo, 135-8550 Japan; 7 Department of Pharmacy, Cancer Institute Hospital, Japanese Foundation for Cancer Research, 3-8-31 Ariake, Koto-ku, Tokyo, 135-8550 Japan; 8 Department of Infectious Diseases, Fujita Health University School of Medicine, 1-98 Dengakugakubo, Kutsukake-cho, Toyoake, Aichi 470-1192 Japan; 9 Division of Infectious Diseases, University of Pittsburgh School of Medicine, 3550 Terrace Street, Pittsburgh, PA 15261 USA

**Keywords:** *Enterobacter cloacae* complex, IMP-type carbapenemase, Outbreak, Whole-genome sequencing, Single nucleotide polymorphism analysis

## Abstract

**Background:**

Information about the clinical and microbiological characteristics of IMP-producing *Enterobacterales* has been limited. Here, we describe an institutional outbreak of IMP-producing *Enterobacter cloacae* complex (ECC) involving multiple clades of ECC sequence type (ST) 78 strains.

**Methods:**

Antimicrobial susceptibility testing, whole-genome sequencing, and conjugation experiments of 18 IMP-producing ECC strains isolated during four-year study period were performed. Species and subspecies were determined by average nucleotide identity analysis and clonal relatedness of the isolates was analyzed with multilocus sequence typing and core-genome single nucleotide polymorphism (SNP) analysis. Relevant clinical information was extracted from medical records.

**Results:**

Fourteen of 18 IMP-producing ECC isolates were determined as *Enterobacter hormaechei* ST78. Sixteen isolates, including 13 isolates belonging to ST78, carried *bla*_IMP-1_ in In316-like class 1 integron and also carried IncHI2 plasmids. Conjugation experiments were successful for 12 isolates carrying *bla*_IMP-1_ on IncHI2 plasmids and for an isolate carrying *bla*_IMP-11_ on an IncL/M plasmid. Although isolation of ST78 strains was clustered in a 14-months period suggesting nosocomial transmission, these strains were subdivided into three clades by SNP analysis: clade A (*n* = 10), clade B (*n* = 1), clade C (*n* = 3). A part of clonal relatedness was unexpected by the epidemiological information at the time of isolation of the strains. Most of the IMP-producing ECC strains were susceptible to non-β-lactam antibiotics and had relatively low minimum inhibitory concentrations to carbapenems (≤4 μg/mL). Five of six infections caused by IMP-producing ECC were treated successfully.

**Conclusions:**

Whole-genome sequencing analysis revealed the outbreak was caused by three different clades of ST78 strains, where patients had favorable treatment outcome of the infections compared with that caused by *Enterobacterales* producing other carbapenemases, possibly due to their non-multidrug-resistant phenotype.

**Supplementary Information:**

The online version contains supplementary material available at 10.1186/s12879-021-05952-9.

## Background

Carbapenem-resistant *Enterobacterales* (CRE) has been spreading globally during the last decade and acknowledged as an imminent risk for public health due to the limited treatment options for the infections caused by the organisms [1]. Among the various mechanisms of carbapenem resistance in *Enterobacterales*, production of carbapenemases is clinically the most important. Carbapenemase-producing *Enterobacterales* (CPE) may have a higher risk of detrimental outcomes from invasive infections and of spreading resistance genes in healthcare facilities by clonal expansion or conjugative transfer of resistance plasmids compared with non-carbapenemase-producing CRE [2, 3]. KPC enzymes belonging to Ambler class A, IMP, VIM, NDM enzymes (metallo-β-lactamases: MBLs) belonging to Ambler class B, and OXA-48-group enzymes belonging to Ambler class D are the major carbapenemases produced by *Enterobacterales*. Although KPC enzymes are the most common globally, epidemiology of carbapenemases produced by *Enterobacterales* differs in each region and country of the world [4].

Carbapenem resistance among *Enterobacterales* clinical strains has been relatively infrequent in Japan. A national surveillance conducted by National Institute of Infectious Diseases reported that resistance rates to meropenem of *Escherichia coli*, *Klebsiella pneumoniae*, and *Enterobacter cloacae* complex (ECC) in 2018 were 0.1, 0.5, and 1.1%, respectively (https://janis.mhlw.go.jp/english/report/open_report/2018/3/1/ken_Open_Report_Eng_201800_clsi2012.pdf). Although IMP enzymes have been overwhelmingly dominant among carbapenemases produced by *Enterobacterales* in Japan, major species of CPE is different in each geographic area in Japan. While IMP-producing ECC is most common in Tokyo, IMP-producing *K. pneumoniae* and *E. coli* are more common around Osaka [5, 6].

While transmission dynamics of KPC-producing *Enterobacterales* in healthcare facilities have been reported abundantly in the literature, information about transmission of IMP-producing *Enterobacterales* is scarce [7–9]. Although a recent study demonstrated that mortality of patients with isolation of IMP-producing *Enterobacterales* was similar to that of patients with isolation of non-CPE, detailed characteristics of infections caused by IMP-producing *Enterobacterales* remain to be clarified [10]. Although recent global clinical studies have addressed treatment of infections caused by CPE, few cases of infections caused by IMP-producing *Enterobacterales* were included due to their rarity [11].

Starting in July 2014, an outbreak of IMP-producing ECC occurred at a cancer center in Tokyo, Japan. Here, we report the clinical characteristics of infections caused by IMP-producing *Enterobacterales* and the results of microbiological and molecular analysis to infer the route of transmission.

## Methods

### Setting and design

This is a descriptive study of an institutional outbreak of CPE at a 700-bed cancer center in Tokyo, Japan. It provides care for patients with all type of malignancies. Annually, 17,500 patients are hospitalized with a mean of 12 days. All the CPE isolates between January 2014 and December 2017 were analyzed. In addition, clinical and epidemiological investigation was performed for all the patients who had CPE isolated.

### Clinical data collection

Clinical information of the patients with CPE was extracted from the medical records retrospectively and included age, sex, type of malignancy and other comorbidities, date of the first isolation of CPE, type of sample from which CPE was isolated, hospitalized ward, department caring the patient, clinical significance of the CPE isolates (infection or colonization), use of antimicrobial agents within 30 days prior to the isolation of CPE, type and date of surgery within 90 days prior to the isolation, type of endoscopy within 90 days prior to the isolation, cancer chemotherapy within 90 days prior to the isolation, admission to the hospital during the study period and to other hospitals within a year prior to the isolation, and death within 30 and 90 days after the isolation of CPE. Additionally, the type of infection, antimicrobial treatment, necessity and achievement of source control, and prognosis were reviewed for the cases with infections caused by CPE. Infection and colonization were determined according to the CDC definition [12].

### Identification of carbapenemase-producing *Enterobacterales* at the hospital

Routine bacterial identification and antimicrobial susceptibility testing were performed with MicroScan WalkAway (Beckman Coulter, Brea, CA, USA) at the hospital. The results of antimicrobial susceptibility testing were interpreted with CLSI M100-S17 guidelines in 2014, and with CLSI M100-S22 guidelines, in which lower breakpoints of cephalosporins and carbapenems for *Enterobacterales* were adopted, from 2015 through 2017 [13, 14]. Testing for carbapenemase production was performed on the isolates showing non-susceptibility against cefepime or any carbapenems. Non-susceptibility to cefepime was added to the screening criteria due to concerns about low sensitivity of the screening using non-susceptibility to carbapenems alone for the detection of IMP-producing *Enterobacterales* [15]. Carbapenemase production (focusing on MBLs) was confirmed with ceftazidime 30-μg disks (Eiken Chemical, Tokyo, Japan), imipenem 10-μg disks (Eiken Chemical, Tokyo, Japan), and sodium mercaptoacetate (SMA) 3-mg disks (Eiken Chemical, Tokyo, Japan). Isolates showing enlargement of inhibitory zone diameters around the ceftazidime disk or imipenem disk by > 5 mm when it was located adjacent to an SMA disk were determined to be carbapenemase-producing *Enterobacterales* [16, 17]. Additionally, modified carbapenem inactivation method (mCIM) according to CLSI M100-S27 guidelines was performed after April 2017 [18].

### Active surveillance culture for CPE

A ward-wide active surveillance for multidrug-resistant *Enterobacterales* was conducted for the selected wards intermittently during the study period. All patients except those hospitalized less than 3 days were included. Stool or rectal swab samples were obtained. If patients had urinary catheters placed, urine was collected. Because epidemiological investigation suggested possible transmission associated with respiratory procedures among post-surgical patients in Surgery-B department, throat swab was also collected from those patients. Culture was performed using CHROMagar ESBL (Kanto Chemical, Tokyo, Japan). Antimicrobial susceptibility testing was performed on the isolates grown on the agar and CPE was identified with the same protocol as clinical culture.

### Microbiological and molecular analysis

All CPE isolates detected first from each patient at the hospital were collected and analyzed at a research laboratory. Because susceptibility testing of carbapenems with drug concentration below 4 μg/mL was not performed at the hospital in 2014, antimicrobial susceptibility testing of all isolates was conducted again at the research laboratory with BD Phoenix NMIC/ID-208 panel (BD Diagnostics, Sparks, Maryland, USA) and results were interpreted according to CLSI M100-S27 guidelines [18]. Carriage of *bla*_IMP-1-group_ genes was screened by PCR as described previously [19]. Whole genome sequencing was performed with Illumina Miseq (Illumina, San Diego, California, USA). Genomic DNA libraries were prepared with Nextera XT DNA library preparation kit (Illumina) and were sequenced for 600 cycles (300-bp paired-end reads). Raw reads generated by Miseq were quality trimmed with Trimmomatic tool (version 0.38) and assembled using SPAdes (version 3.12.0). Since all CPE isolates were identified as ECC or related species by MicroScan WalkAway at the hospital, species and subspecies were determined by comparing the average nucleotide identity (ANI) of the genomes of study isolates with those of type strains of ECC using a threshold of > 96.5% at ANI Calculator of EZ BioCloud website (https://www.ezbiocloud.net/tools/ani) [20]. Multilocus sequence typing (MLST), plasmid replicon typing, plasmid MLST, and screening of acquired resistance genes were conducted with MLST 1.8, PlasmidFinder 1.3, pMLST 1.4, and ResFinder 3.0, respectively, at Centers for Genomic Epidemiology website (https://cge.cbs.dtu.dk/services/). Structures of integrons were analyzed with Basic Local Alignment Search Tool (BLAST) (https://blast.ncbi.nlm.nih.gov/Blast.cgi) using nucleotide sequences of contigs containing *bla*_IMP-1-group_. Core-genome single nucleotide polymorphism (SNP)-based phylogenetic analysis of ST78 isolates of ECC was performed as described previously using genomic sequence of *E. cloacae* strain 109 belonging to ST78 (GenBank accession number NZ_CP020525) as the reference [6]. Conjugation experiments were carried out with filter mating methods using non-lactose-fermenting *E. coli* ML4909 strain (a rifampin-resistant mutant derived from *E. coli* K-12) as a recipient. Transconjugants were selected on Drigalski lactose agar supplied with moxalactam (16 μg/mL) and rifampin (100 μg/ml). The conjugation experiment was attempted three times for each donor isolate. Carriage of *bla*_IMP-1-group_ were confirmed with PCR and PCR-based replicon typing of the plasmids were performed for transconjugants [21].

### GenBank accession number

All nucleotide sequences of draft genomes have been deposited in the NCBI database under BioProject accession number PRJDB9939.

## Results

### Outbreak description

**Fig. 1:**
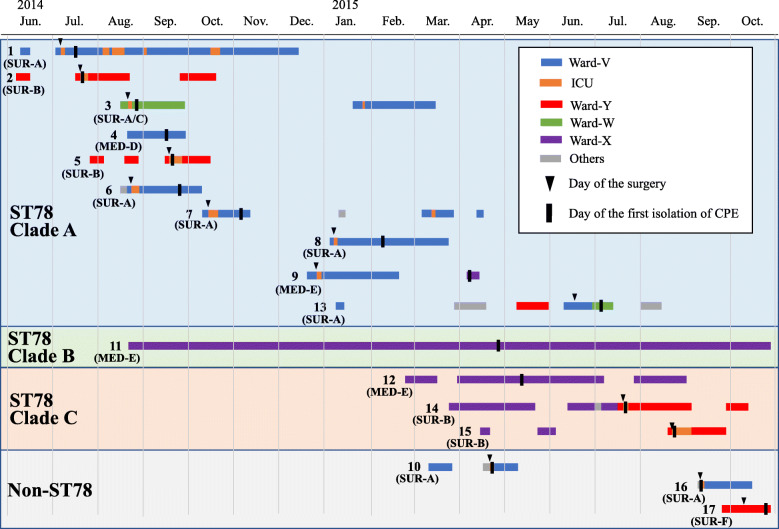
Timeline of hospitalization, surgery, and first isolation of carbapenemase-producing *Enterobacterales* of the patients. The numbers located at the left end of the bars represent Patient ID. The departments caring the patients at the time of isolation of carbapenemase-producing *Enterobacterales* were presented below the Patient ID. SUR, Surgery; MED, Medical Oncology

In July 2014, CPE was detected from a hospitalized patient at the cancer center for the very first time. In total, 18 hospitalized patients were found to have CPE during the study period confirmed by clinical culture (*n* = 13) or surveillance culture (*n* = 5) (Table 1, Table S1). Seventeen and one isolates were identified as *E. claocae* and *Cronobacter sakazakii*, respectively, with MicroScan WalkAway. All isolates were positive for production of MBLs by the SMA disk testing. Date of first isolation of CPE from all but one (Patient-18) patients was clustered in a 16-months period (between July 2014 and Oct 2015). The mean age of 18 patients was 68.8 years. Sixteen patients (88.9%) were male. Seventeen patients (94.4%) had gastrointestinal malignancy. Six (33.3%), fourteen (77.8%), and sixteen (88.9%) patients had received chemotherapy within 90 days, surgery within 90 days, and antimicrobial therapy within 30 days of the first isolation of CPE, respectively. Although the patients were under the care of 7 different departments, Surgery-A (*n* = 7), Surgery-B (*n* = 4), and Medical Oncology-E (*n* = 3) departments were involved in the care of multiple patients. At the time of CPE isolation, the patients were located at one of five different wards or one intensive care unit (ICU). Six, five, and three patients were at ICU, Ward-V, and Ward-X, respectively. Fifteen patients (83.3%) received endoscopic procedure within 90 days of isolation of CPE. All six patients from whom CPE was isolated within 3 days after surgery (Patient-2, − 5, − 10, − 14, − 15, and − 16) received routine airway nebulization and frequent airway suctioning at ICU and bronchoscopy was also performed at operating rooms in two patients (Patient 5 and − 15). Thirteen (76%) of seventeen case patients between July 2014 and Oct 2015 had history of hospitalization at multiple wards before the isolation of CPE (Fig. 1).

**Table 1:** Clinical characteristics of patients from whom carbapenemase-producing *Enterobacterales* strains were isolated

Characteristics	All patients (*n* = 18)	Patients with isolation of ECC ST78 (*n* = 14)	Patients with isolation of ECC non-ST78 (*n* = 4)
Age, mean y [SD]	68.8 [8.1]	69.1 [7.7]	67.8 [9.0]
Age > 65y	12 (66.7)	9 (64.3)	3 (75)
Sex			
Male	16 (88.9)	13 (92.9)	3 (75)
Female	2 (11.1)	1 (7.1)	1 (25)
Status			
Infection	6 (33.3)	5 (35.7)	1 (25)
Colonization detected by clinical cultures	7 (38.9)	4 (28.6)	3 (75)
Colonization detected by surveillance cultures	5 (27.8)	5 (35.7)	0 (0)
Sample from which CPE was first isolated			
Sputum	6 (33.3)	5 (35.7)	1 (25)
Bile	3 (16.7)	0 (0)	3 (75)
Stool	3 (16.7)	3 (21.4)	0 (0)
Intraabdominal fluid	2 (11.1)	2 (14.3)	0 (0)
Other	4 (22.2)	4 (28.6)	0 (0)
Ward at the time of isolation of CPE			
ICU	6 (33.3)	4 (28.6)	2 (50)
Ward-V	5 (27.8)	5 (35.7)	0 (0)
Ward-X	3 (16.7)	3 (21.4)	0 (0)
Other	4 (22.2)	2 (14.3)	2 (50)
Department at the time of isolation of CPE			
Surgery-A	7 (38.9)	5 (35.7)	2 (50)
Surgery-B	4 (22.2)	4 (28.6)	0 (0)
Medical Oncology-E	3 (16.7)	3 (21.4)	0 (0)
Other	4 (22.2)	2 (14.3)	2 (50)
Type of malignancy			
Gastrointestinal	17 (94.4)	14 (100)	3 (75)
Thoracic	1 (5.6)	0 (0)	1 (25)
Cancer chemotherapy within 90 days	6 (33.3)	6 (42.9)	0 (0)
Antimicrobial use within 30 days	16 (88.9)	13 (92.9)	3 (75)
Surgery within 30 days	14 (77.8)	10 (71.4)	4 (100)
Gastrointestinal	13 (72.2)	10 (71.4)	3 (75)
Thoracic	1 (5.6)	0 (0)	1 (25)
Endoscopic procedure within 90 days^a^	15 (83.3)	12 (85.7)	3 (75)
Laryngoscopy	3 (16.7)	3 (21.4)	0 (0)
Esophagogastroscopy	13 (72.2)	11 (78.6)	2 (50)
Duodenoscopy with ERCP	4 (22.2)	2 (14.3)	2 (50)
Colonoscopy	6 (33.3)	5 (35.7)	1 (25)
Bronchoscopy	2 (11.1)	2 (14.3)	0 (0)
30-day mortality	1 (5.6)	1 (7.1)	0 (0)
90-day mortality	3 (16.7)	2 (14.3)	1 (25)

Data are mean [SD] or n (%)

^a^ If a patient received multiple endoscopic procedure, all were counted separately

*ECC Enterobacter cloacae* complex, *CPE* carbapenemase-producing *Enterobacterales*, *ERCP* endoscopic retrograde cholangiopancreatography

A ward-wide active surveillance was performed twice at Ward-V (November 2014 and February 2015), three times at Ward-X (May, June, and July 2015), and once at Ward Y (November 2014). A total of 191 patients were screened and two patients were positive for carriage of CPE (Patient-7 and -12). Additionally, a patient with a history of admission in Ward-V was screened upon a later admission at Ward-X and was positive for the growth of CPE (Patient-9). Surveillance cultures performed at the discretion of primary physicians immediately after surgery without using selective media turned positive for carriage of CPE in two patients (Patient-2 and -6).

Six patients had infections due to CPE (Table 2). Although one patient died of an intraabdominal infection following intestinal perforation on the 11th day of the onset of the infection, infections in other patients were cured without relapse. Two other patients died within 90 days due to reasons unrelated to the carriage of CPE (Table S1).

**Table 2:** Clinical characteristics and outcomes of infections caused by carbapenemase-producing *Enterobacterales* strains

Patient No.	Diagnosis of the infectious diseases	Setting	Samples positive for CPE	Adequate source control	Antimicrobial treatment^a^	Prognosis	Comment
1	Cholangitis Liver abscess	After resection of gastrointestinal malignancy and choledochoduodenostomy	Blood (Day 1, Day 28) Drained abscess (Day 29)	Established (percutaneous abscess drainage)	(Day 1–3) FEP (IV) (Day 3–8) MEM (IV) + GEN (IV) (Day 9–28) MEM (IV) (Day 29–30) MEM + GEN (IV) + LVX (IV) (Day 31–43) MEM (HD-EX, IV) + GEN (IV) + LVX (IV) (Day 44–74) TZP (HD-EX, IV) + GEN (IV) + LVX (IV)	Cure without 2nd relapse	Cholangitis with bacteremia due to CPE developed 9 days after surgery was treated with MEM (Day 3–28) according to the susceptible result at the hospital. Although fever and bacteremia were once resolved, they recurred on Day 28. CT scan of the abdomen revealed liver abscess. Percutaneous abscess drainage was performed and antimicrobial treatment was re-initiated. TZP (Day 44–74) was selected according to the susceptible result at the hospital.
3	Surgical site infection (deep incisional)	After resection of gastrointestinal malignancy	Abscess (Day 1)	Established (incision and drainage)	(Day 1–9) TZP (IV) (Day 9–17) MEM (IV) (Day 17–25) LVX (PO)	Cure without relapse	Deep incisional surgical site infection developed 5 days after surgery. MEM (Day 9–17) was selected according to the susceptible result at the hospital.
4	Intraabdominal infection	Under palliative care for advanced gastrointestinal malignancy	Intraabdominal fluid (Day 1)	Unestablished	(Day 1–6) MEM (IV) (Day 6–11) IPM (IV)	Death (Day 11)	Although intestinal perforation was suspected by imaging studies, surgical intervention was not performed because the patient was on do-not-resuscitate order due to his advanced cancer. IPM (Day 6–11) was selected according to the susceptible result at the hospital.
8	Intraabdominal infection	After resection of gastrointestinal malignancy	Intraabdominal fluid (Day 1)	Established (percutaneous fluid drainage)	(Day 1–7) AMC (PO)	Cure without relapse	An intraabdominal fluid collection was found on abdominal CT scan 34 days after surgery. Percutaneous fluid drainage was performed and the culture of fluid grew CPE. A low-grade fever subsided after the percutaneous fluid drainage.
15	Pneumonia	After resection of gastrointestinal malignancy	Sputum (Day 1)	Unnecessary	(Day 1–3) SAM (IV) (Day 3–14) MEM (IV) + LVX (IV)	Cure without relapse	High fever and productive cough developed on the next day of surgery, A new pulmonary infiltrate was found on chest X-ray.
16	Surgical site infection (deep incisional and intraabdominal space)	After resection of gastrointestinal malignancy	Blood (Day 1) Abscess (Day 1) Intraabdominal fluid (Day 3)	Established (incision and drainage of the wound surface, and percutaneous peritoneal drainage)	(Day 1–3) MEM (IV) + GM (IV) (Day 3–21) MEM (HD-EX, IV) + GEN (IV) (Day 22–24) SXT (PO) (Day 24–38) LVX (PO)	Cure without relapse	CPE was first identified from surveillance bile culture during the surgery prior to the onset of infection. SXT was changed to LVX (Day 24) due to the possible side effect of nausea.

^a^ Only antimicrobial agents with activity gram-negative organisms were presented. Antimicrobial agents against which the causative organisms were susceptible by antimicrobial susceptibility testing with BD Phoenix NMIC/ID-208 panel interpreted with CLSI M100-S27 guidelines were underlined

*FEP* cefepime, *MEM* meropenem, *GEN* gentamicin, *LVX* levofloxacin, *TZP* piperacillin-tazobactam, *IPM* imipenem, *AMC* amoxicillin-clavulanic acid, *SAM* ampicillin-sulbactam, *SXT* trimethoprim-sulfamethoxazole, *HD-EX* high-dose and extended infusion, *IV* intravenous, *PO* oral

### Outbreak management

The patients with a history of isolation of CPE were cared for in a private room under contact precautions according to the infection prevention protocol of the hospital. After July 2014 when the isolation of CPE from patients hospitalized in different wards was documented, occurrence of an institutional outbreak of CPE was notified to the all hospital staffs and strict compliance to the infection prevention protocol was enforced. In addition, direct observation of hand hygiene compliance was initiated by infection preventionists and the data were fed back to each hospital department. Compliance to the infection prevention protocol was thoroughly checked especially at the wards where the patients with CPE was hospitalized. Sampling from hospital environment was not performed. Compliance to the cleaning and disinfection protocol of endoscopes was confirmed and bacterial cultures of the relevant endoscopes, including bronchoscopes for the operating rooms and ICU and duodenoscope, were negative for the growth of CPE.

### Microbiological and molecular analysis of CPE

All CPE isolates were non-susceptible to cefepime and all but one isolates were non-susceptible to piperacillin-tazobactam with BD Phoenix NMIC/ID-208 panel. Three isolates were susceptible to aztreonam. Although most of the isolates were non-susceptible to carbapenems, MIC of > 4 μg/mL for imipenem and meropenem was observed only in two isolates and one isolate, respectively. Most of the isolates was susceptible to non-β-lactam antibiotics tested (Table 3).

**Table 3:** Molecular and microbiological characteristics of carbapenemase-producing *Enterobacterales* isolates

Patient No.	Isolate name	Date of isolation	Bacterial species of CPE (ANI)^a^	Sequence type	Clade^b^	Antimicrobial susceptibility^c^							MIC (mg/mL)^d^		Antimicrobial resistance genes	Plasmid replicons^e^
						TZP	FEP	ATM	LVX	SXT	GEN	AMK	IPM	MEM		
1	TUM 14647	Jul. 2014	*E. hormaechei*	78	A	R	R	R	S	S	S	S	4	4	*bla*_IMP-1_*, bla*_ACT-5_*, aac(6′)-IIc, qnrB6, fosA, sul1, tet(B)*	IncHI2A/IncHI2
2	TUM 14648	Jul. 2014											2	4		
3	TUM 14652	Aug. 2014											4	4		
4	TUM 14654	Sep. 2014											4	2		
5	TUM 14658	Sep. 2014											4	2		
6	TUM 14792	Sep. 2014											4	2		
7	TUM 14797	Nov. 2014											4	4		
8	TUM 17939	Feb. 2015											4	2		IncHI2A/IncHI2
9	TUM 17940	Apr. 2015				I	R	I	I	R	S	S	> 4	> 4		
13	TUM 17944	Jul. 2015				R	R	R	S	S	S	S	4	2		
11	TUM 17942	Apr. 2015			B	R	SDD	S	R	R	S	S	4	≤1	*bla*_IMP-11_*, bla*_TEM-1B_*, bla*_ACT-5_*, aac(6′)-Ia, qnrS1, fosA, sul1*	IncFIB (pHCM2) IncL/M (pMU407)
12	TUM 17943	May 2015			C	R	R	R	S	S	S	S	> 4	4	*bla*_IMP-1_*, bla*_ACT-5_*, aac(6′)-IIc, aph(3″)-1b, aph(6)-1d, qnrB6, fosA, sul1, sul2, tet(B)*	IncHI2A/IncHI2
14	TUM 17945	Jul. 2015											4	4		
15	TUM 17946	Aug. 2015											4	4		
10	TUM 17941	Apr. 2015	*E. asburiae*	24	NA	R	R	S	S	S	S	S	4	4	*bla*_IMP-1_*, bla*_ACT-1_*, aac(6′)-IIc, fosA, sul1*	IncFII (pECLA)/ IncFIB (pECLA)
16	TUM 17947	Sep. 2015	*E. xiangfangensis*	1331	NA	R	R	R	S	S	S	S	4	4	*bla*_IMP-1_*, bla*_ACT-16_*, aac(6′)-IIc, strA, aph(6)-1d, qnrS1, fosA, sul1, tet(B)*	IncHI2A/IncHI2 IncFIB (pECLA)
17	TUM 17948	Oct. 2015	*E. hormaechei subsp. steigerwaltii*	113	NA	S	R	S	S	S	S	S	4	2	*bla*_IMP-1_*, bla*_ACT-15_*, aadA1, aac(6′)-IIc, qnrB6, fosA, sul1*	IncHI2A/IncHI2 IncFII (pECLA)/ IncFIB (pECLA)
18	TUM 17949	Apr. 2017	*E. hormaechei subsp. steigerwaltii*	133	NA	R	R	S	I	S	S	S	2	≤1	*bla*_IMP-1_*, bla*_ACT-7_*, aac(6′)-IIc, qnrB6, fosA, sul1, tet(B)*	IncHI2A/IncHI2

^a^ Bacterial species and subspecies confirmed by the comparison of average nucleotide identity of study isolates with those of type strains were presented

^b^ Clades of ST78 isolates determined by core-genome single nucleotide polymorphism-based analysis were presented

^c^ Results of antimicrobial susceptibility testing performed with BD Phoenix NMIC/ID-208 panel and interpreted according to CLSI M100-S27 guidelines were shown

^d^ Minimum inhibitory concentrations determined with BD Phoenix NMIC/ID-208 panel were presented

^e^ Plasmid replicon was underlined if a transconjugant of the isolates was positive for the replicon by PCR-based replicon typing

*ANI* average nucleotide identity, *NA* Not applicable, *ST* sequence type, *TZP* piperacillin-tazobactam, *CTX* cefotaxime, *CAZ* ceftazidime, *FEP* cefepime, *ATM* aztreonam, *IPM* imipenem, *MEM* meropenem, *LVX* levofloxacin, *SXT* trimethoprim-sulfamethoxazole, *GEN* gentamicin, *AMK* amikacin, *S* Susceptible, *SDD* Susceptible-Dose Dependent, *I* Intermediate, *R* Resistant

Fourteen of eighteen CPE isolates were identified as *E. hormaechei* by ANI and other isolates were *E. hormaechei* subsp. *steigerwaltii* (*n* = 2), *E. asburiae* (*n* = 1), and *E. xiangfangensis* (*n* = 1) (Table 3). All *E. hormaechei* isolates were ST78 and isolated between July 2014 and August 2015. While one isolate (TUM17942) carried *bla*_IMP-11_, all other isolates carried *bla*_IMP-1_. As expected, all isolates carried chromosomal *ampC* genes, but acquired genes for extended-spectrum β-lactamases (ESBL), AmpC, and carbapenemases other than *bla*_IMP_ were not identified in any isolates. Plasmid replicon for IncHI2 was documented in 16 isolates and six of these (TUM14648, TUM14652, TUM17945, TUM17946, TUM17947, TUM17949) were pMLST-ST1 (*smr0119*:1-*smr0018*:1). While remaining 10 isolates also had allele 1 of *smr00119*, *smr00118* had 0.3% difference from allele 1 in four isolates (TUM14647, TUM17939, TUM17943, TUM17944), were not fully sequenced in two isolates (TUM14654. TUM17940), and were non-typable in four isolates (TUM14658, TUM14792, TUM14797, TUM17948).

All isolates carrying *bla*_IMP-1_ had In316 (*intI1*-*bla*_IMP-1_-*aac(6′)-IIc*-*sul1*)-like structure. In 7 isolates (TUM17941, TUM17943, TUM17945, TUM17946, TUM17947, TUM17948, and TUM17949), nucleotide sequences of In316 were completely preserved and other isolates had single nucleotide difference (*n* = 5) or had fragmentation of the structure into multiple contigs (*n* = 5).

**Fig. 2:**
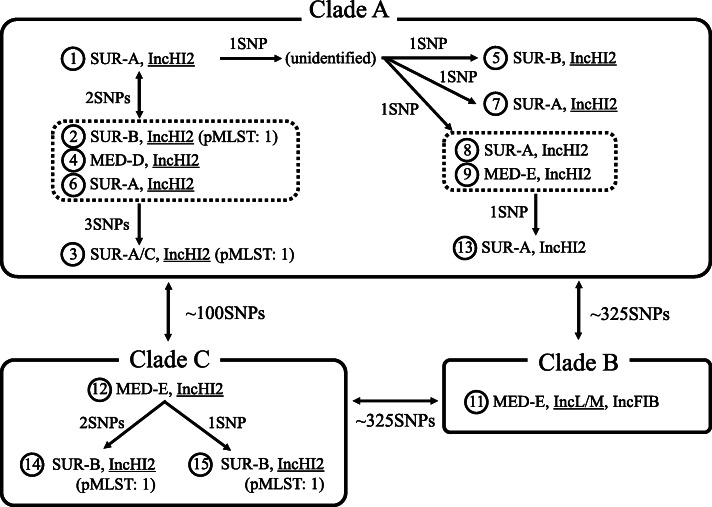
Molecular relatedness of the ST78 isolates from the patients. Isolates were indicated as circles with the patient designation numbers. The departments caring the patients at the time of isolation of ST78 isolates and plasmid replicons of the isolates identified by whole-genome sequencing analysis were shown. Plasmid replicon was underlined if a transconjugant of the isolates carrying *bla*_IMP_ was positive for the replicon by PCR-based replicon typing. pMLST profiles were also shown for the isolates with confirmed results. SNP deference of the isolates from the patients were presented and isolates without SNP difference were enclosed in dotted lines. Isolates belonging to the same clade were enclosed in solid lines. SUR, Surgery; MED, Medical Oncology

ST78 isolates were divided into three clades by core-genome (4,257,370 bp, 82.8% of the genomic sequence of the reference strain) based SNP analysis (Fig. 2). All patients from whom isolates of clade A were identified had a history of admission at ICU, Ward-V, or Ward-Y. On the other hand, all patients from whom isolates of clade C were identified had a history of multiple admission at Ward-X (Fig. 1).

### Conjugation experiments

In 13 isolates, *bla*_IMP_ was successfully transferred into recipient *E. coli* cell by conjugation experiment (Table 3). All transconjugant were positive for PCR using primers for *bla*_IMP-1-group_. TUM17942 yielded transconjugants positive for IncL/M by PCR-based replicon typing. Transconjugants of the remaining isolates were positive for IncHI2 by PCR-based replicon typing, which suggested the location of *bla*_IMP-1_ on IncHI2 plasmids.

## Discussion

During the study period, carriage of CPE by 18 patients was identified. Molecular analysis of the isolates demonstrated an institutional outbreak of IMP-producing ECC ST78 which occurred in 14 patients over 14-months across multiple wards and departments. Core genome-based SNP analysis unexpectedly revealed that the outbreak involved three clades of ST78 isolates suggesting multiple introductions and routes of spread.

ST78 has been recognized as one of the global resistant clones of ECC [22]. ST78 isolates carrying *bla*_KPC_, *bla*_VIM_, and *bla*_OXA-48-group_ have been reported, together with the isolates with *bla*_IMP_ [22–24]. We previously reported that multiple clades of ST78 isolates carrying *bla*_IMP-1_ on IncHI2 plasmids had spread in Tokyo [6]. In this study, we found that multiple clades of ST78 were indeed involved during 16-months outbreak within a single institution. Epidemiological investigation in combination with conventional microbiological analysis was insufficient to elucidate the details of the outbreak, and use of whole-genome sequencing-based analysis, with its resolution to differentiate similar clones, was crucial as described in other outbreaks [7–9].

Two ancestral isolates of clade A (isolates from Patient-1 and -2) had two SNPs differences but no common ancestor of these isolates was identified (Fig. 2). Nevertheless, the time of introduction of the original isolate of clade A was expected to be within a few months prior to the first identification of IMP-producing ECC at this hospital (July 2014), considering that average substitution rate of IMP-producing ST78 isolates in Tokyo was 4.53 SNPs/genome/year in the previous study [6]. Clade A isolates were detected from 10 patients during one-year period, all of whom had a history of hospitalization at ICU, Ward-V, or Ward-Y (Fig. 1). We hypothesized that these isolates were transmitted through medical care either via medical device, environment, or healthcare workers during hospitalization in these wards. While Patient-4 had history of hospitalization only at Ward-V, Patient-2, − 3, − 5 had no history of hospitalization at Ward-V prior to the isolation of clade A isolates. Therefore, involvement of multiple wards in the transmission of clade A isolates was suggested.

An isolate of clade B was isolated only from Patient-11 and carried *bla*_IMP-11_ on an IncL/M plasmid. IMP-11-producing ECC isolates harboring IncL/M plasmids have been reported from Japan [10]. In addition, this patient had a history of recent long-term hospitalization at another hospital. Therefore, acquisition of the clade B isolate likely occurred outside the cancer center.

Clade C isolates were detected from three patients who had been all hospitalized in Ward-X during the same period. IMP-producing isolates were identified from the sputum of Patient-14 and -15 on the next day of the surgery performed by the same department (Surgery-B) at ICU, thus acquisition through respiratory procedure at ICU or bronchoscopy at operating rooms was suspected based on epidemiological information (Table S1). However, the whole-genome sequencing analysis revealed that Patient-12 was the index patient and transmission to Patient-14 and 15 most likely occurred during the hospitalization at Ward-X several months prior to the surgery. Cancer patients often require multiple hospitalizations at different wards and are managed by different departments including surgery, radiology, and medical oncology during a long course of treatment. This complexity of care makes it very challenging to infer route of transmission of resistant organisms based on epidemiological investigation alone. Real-time performance of whole-genome sequencing analysis would be more useful in the investigation of these outbreaks involving patients requiring complex medical care.

Patient-10, − 16, − 17, and − 18 carried IMP-producing non-ST78 ECC isolates, which suggests these patients incidentally acquired the isolates unrelated to the outbreak. Notably, two non-ST78 isolates (TUM17947 and TUM17948) carried *bla*_IMP-1_ on IncHI2 plasmids. Carriage of *bla*_IMP-1_ on IncHI2 plasmids by ECC isolates of multiple STs (e.g., ST53, 78, 113, 513, 1047) has been documented in another area (Nagoya) in Japan [25]. Although ST78 isolates appears to predominate among IMP-producing ECC isolates in Tokyo according to our previous study, the nationwide epidemiology remains to be elucidated [6]. Although there is the possibility of conjugative transfer of IncHI2 plasmids carrying *bla*_IMP-1_ between ST78 isolates and non-ST78 isolates in the hospital environment or in the flora of the patients, it is beyond the scope of our analysis.

In this study, most of the IMP-producing ECC isolates were susceptible to non-β-lactam antibiotics and had relatively low MICs (≤4 μg/mL) to carbapenems. Furthermore, several isolates were susceptible to non-carbapenem β-lactams such as piperacillin-tazobactam and aztreonam. These patterns of antimicrobial susceptibilities were similar to the IMP-producing *Enterobacterales* in Japan in previous reports [6, 10]. Infections caused by IMP-producing ECC were treated successfully mainly with non-β-lactam antibiotics retaining activity to the organisms except a case of intraabdominal infection without adequate source control (Table 2). Better prognosis of infections caused by MBL-producing *Enterobacterales* associated with better antimicrobial susceptibilities compared with those caused by KPC-producing *Enterobacterales*, which was consistent with our observation, was reported in an observational study [26].

Our study has several limitations. First, there could have been missed patients carrying CPE in the outbreak for several reasons. Active surveillance cultures were performed for a limited number of times for selected wards only. In addition, selective media for ESBL, not specific for CPE, was used for surveillance culture. Although previous studies showed that selective media for ESBL had > 90% sensitivity for the isolation of CPE as a whole, the ability to identify IMP-producing isolates was unclear [27]. Screening criteria for the routine culture testing for the performance of confirmation testing for carbapenemase production were not strict enough to identify all CPE isolates. However, the lack of isolation of IMP-producing ECC clonal isolates for more than 2 years after August 2015 suggests successful containment of major transmissions with reinforcement of compliance to infection prevention protocols including hand hygiene practice. Second, we have not identified the direct route of introduction and transmission of IMP-producing ECC isolates. Apparent common sources were not identified by clinical epidemiological analysis and surveillance cultures of medical devices such as endoscopes were negative. Third, number of the cases of infections caused by IMP-producing ECC was too limited to analyze the association between the treatment regimen and prognosis.

## Conclusions

Involvement of multiple clades of ST78 isolates was documented by whole-genome sequencing analysis of IMP-producing ECC isolates identified in a hospital-wide outbreak. Genetic relatedness of the isolates unexpected by the clinical analysis was uncovered. Real-time performance of whole-genome sequencing of relevant bacterial isolates in complicated epidemiological situations could facilitate identification transmission route, which is vital in containing outbreaks of antimicrobial-resistant organisms.

## Supplementary Information

**Additional file 1: Table S1.** Characteristics of each patient from whom carbapenemase-producing *Enterobacterales* strains were isolated.

## Data Availability

All data generated or analysed during this study are included in this published article.

## Abbreviations

CRE: Carbapenem-resistant *Enterobacterales*
CPE: Carbapenemase-producing *Enterobacterales*
MBL: Metallo-β-lactamase
ECC: *Enterobacter cloacae* complex
SMA: Sodium mercaptoacetate
mCIM: Modified carbapenem inactivation method
ANI: Average nucleotide identity
MLST: Multilocus sequence typing
BLAST: Basic local alignment search tool
SNP: Single nucleotide polymorphism
ICU: Intensive care unit
ESBL: Extended-spectrum β-lactamases
